# The Dynamic Response of Sweat Chloride to Changes in Exercise Load Measured by a Wearable Sweat Sensor

**DOI:** 10.1038/s41598-020-64406-5

**Published:** 2020-05-07

**Authors:** Dong-Hoon Choi, Grant B. Kitchen, Kerry J. Stewart, Peter C. Searson

**Affiliations:** 1 Institute for Nanobiotechnology, Johns Hopkins University, Baltimore, USA; 2 Department of Medicine, Johns Hopkins University School of Medicine, Baltimore, USA; 3 Department of Materials Science and Engineering, Johns Hopkins University, Baltimore, USA

**Keywords:** Biomedical engineering, Analytical chemistry, Physiology

## Abstract

Wearable sensors enable the monitoring of an individual’s sweat composition in real time. In this work, we recorded real-time sweat chloride concentration for 12 healthy subjects in three different protocols involving step changes in exercise load and compared the results to laboratory-based analysis. The sensor results reflected the changes in exercise load in real time. On increasing the exercise load from 100 W to 200 W the sweat chloride concentration increased from 12.0 ± 5.9 to 31.4 ± 16 mM (mean ± SD). On decreasing the load from 200 W to 100 W, the sweat chloride concentration decreased from 27.7 ± 10.5 to 14.8 ± 8.1 mM. The half-time associated with the change in sweat chloride, defined as the time at which the concentration reached half of the overall change, was about 6 minutes. While the changes in sweat chloride were statistically significant, there was no correlation with changes in sweat rate or other physiological parameters, which we attribute to intra-individual variation (SD = 1.6–8.1 mM). The response to exercise-induced sweating was significantly different to chemically-induced sweating where the sweat chloride concentration was almost independent of sweat rate. We speculate that this difference is related to changes in the open probability of the CFTR channel during exercise, resulting in a decrease in reabsorption efficiency at higher sweat rates.

## Introduction

The most effective method of thermal regulation during exercise is sweating, however, excessive water and electrolyte losses can contribute to dehydration and electrolyte imbalances^1–4^. Measurement of electrolyte loss during sweating usually involves absorption patches, collection devices (e.g. Macroduct), or plastic bags to collect samples to be sent to a laboratory for analysis^4–8^. Since sweat collection times are generally in the range of 5 to 90 minutes, depending on the study^8^, laboratory analysis has limited capability for elucidating real-time, dynamic changes during exercise. Recent advances in wearable sweat sensors^9–13^ have enabled real-time measurement of water and electrolyte loss during exercise. In addition, continuous measurement enables detection of transient changes that could be masked by the sweat collection time in conventional measurements. Finally, wearable sensors require very small volumes for measurement in comparison to standard laboratory-based tests. For example a sweat test for diagnosis of cystic fibrosis requires a minimum sweat volume of 15 µL^14^ whereas a sweat sensor can detect sweat volumes less than 1 μL^15^.

Electrolytes regulate fluid balance in plasma and tissues and are involved in cell signaling^16^, and electrolyte imbalance can lead to a wide range of medical conditions^17,18^. Chloride and sodium are the most abundant electrolytes and their concentrations are closely correlated^19^. In this work, to assess the feasibility of using a wearable sensor to evaluate transient changes in real-time, we performed a systematic study of the dynamic response of sweat chloride to step changes in exercise load. We performed trials with 12 healthy subjects while spinning on a stationary bike using three different protocols involving step changes in power output from 100 W to 200 W, 200 W to 100 W, and 100 W to 200 W in 25 W increments. In addition to sweat chloride, we measured changes in other physiological parameters including heart rate, skin temperature, and local sweat rate. The sensor accuracy was validated by comparing values to conventional laboratory-based analysis of sweat samples collected by a Macroduct device. We show that the changes in sweat chloride (∆C) on changing exercise load were statistically significant, although there was no correlation between ∆C and sweat rate or other physiological parameters, which we attribute to intra-individual variation. We also show that the response to exercise-induced sweating and chemically-induced sweating are significantly different, although values obtained at an exercise intensity below 100 W exercise load are comparable to results from chemically-induced sweating.

## Results

### Sweat profiles measured using a wearable sensor

**Figure 1:**
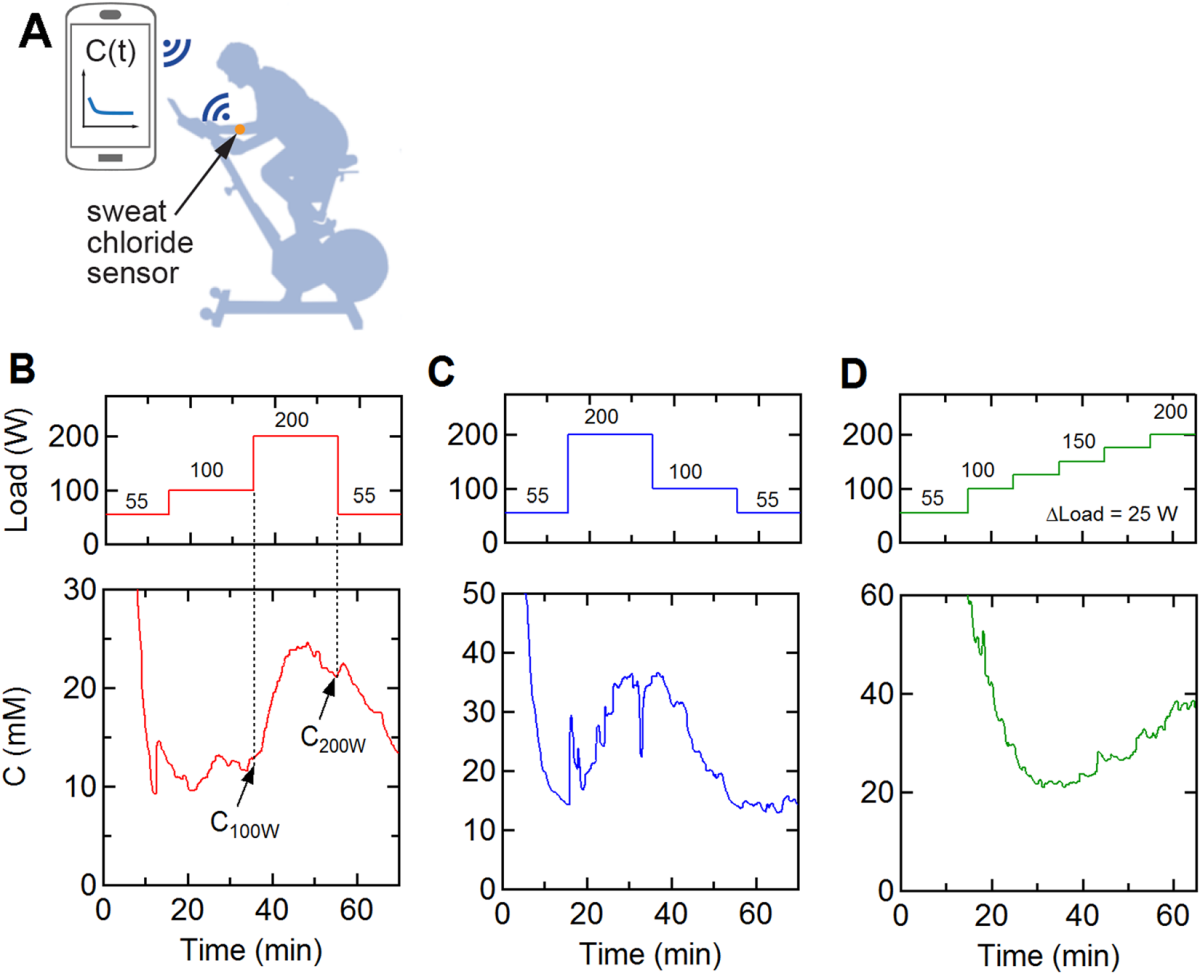
Sweat profiles measured by a wearable sensor. (**A**) Measurement set-up. (**B–D**) Representative sweat chloride profiles at three different exercise protocols. (**B**) Single step trial (100 W for 20 minutes, 200 W for 20 minutes). (**C**) Reverse step trial (200 W for 20 minutes, 100 W for 20 minutes). (**D**) Multi-step trial (100–200 W in 25 W increments each for 10 minutes). All protocols included a 15 minute warm-up at 55 W. The sweat profiles were obtained from the same individual.

12 healthy subjects were asked to spin on a stationary bike following three different protocols following a warm up: (1) a single step trial: 100 W (20 minutes) to 200 W (20 minutes), (2) a reverse step trial: 200 W (20 minutes) to 100 W (10 minutes), and (3) a multi-step trial: 100 W–200 W in 25 W increments for 10 minutes each. During the trials, the sweat chloride concentration was measured using a wearable sweat sensor, Bluetooth transceiver, and smartphone app (Fig. 1A). Typical sweat profiles for one subject recorded across the three protocols are shown in Fig. 1B–D. The initial decrease in sweat chloride concentration during the warm-up was due to the high electrical impedance prior to the onset of sweating and does not reflect a high sweat concentration. The onset of sweating and stabilization of the sensor output occurred after 15–25 minutes. For this subject, in the 100–200 W trial, the sweat chloride concentration was about 12 mM during the 100 W segment but increased to 21 mM in the 200 W segment (Fig. 1B). During the reverse step trial, the sweat concentration increased to 35 mM during the 200 W segment and then decreased to 14.2 mM on decreasing the power output to 100 W (Fig. 1C). During the multi-step trials (Fig. 1D), the concentration increased following the incremental increases in power output. These changes in sweat chloride concentration are related to the associated changes in sweat rate and level of effort^20–26^.

### Changes in sweat chloride concentration on changing exercise load

**Figure 2:**
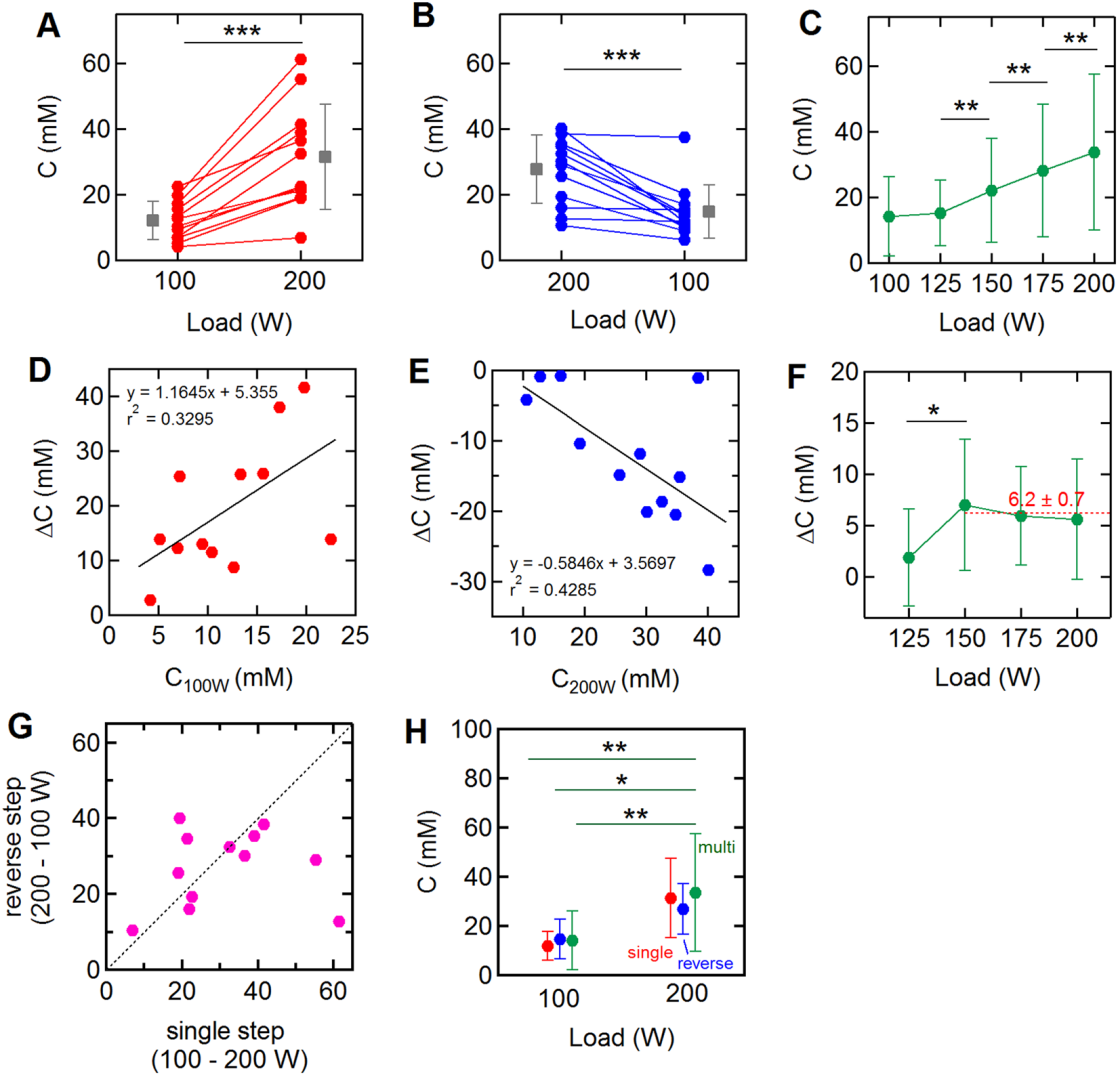
Relation between sweat chloride concentration and exercise load. (**A**–**C**) Change in sweat chloride concentration associated with: (**A**) the single step (100 W – 200 W; N = 12), (**B**) the reverse step (200 W–100 W; N = 12), and (**C**) the multi-step (N = 9 at 100 W, N = 12 at 125 to 200 W) trials. Data represent mean ± SD at each exercise load. (**D-E**) Initial sweat concentration versus change in sweat concentration associated with: (**D**) the single step trial (100 W – 200 W), and (**E**) the reverse step trial (200 W – 100 W). C_100W_ and C_200W_ are the concentrations at the end of 100 W and 200 W segments of the single step and reverse single step trials, respectively. (**F**) Concentration changes during the multi-step trials. (**G**) Sweat chloride concentration at 200 W for the single step and reverse step trials. (**H**) Sweat chloride concentration at 100 W and 200 W for single step, reverse step, and multistep trials. Data represent mean ± SD at each exercise load. *p < 0.05, **p < 0.01, ***p < 0.001.

The changes in sweat chloride concentration in response to changes in exercise intensity were determined for all three protocols (Fig. 2A–C). The sweat chloride concentration at each exercise load was determined from the average value over the last 1 minute of that segment (Fig. 1B). The magnitude of the change in concentration was dependent on the individual. For the single step 100–200 W trials, the average concentration at 100 W was 12.0 ± 5.9 (mean ± SD) and increased to 31.4 ± 16.0 mM at 200 W (p = 0.00015) (Fig. 2A). During the reverse step trial, the average sweat concentration decreased from 27.7 ± 10.5 to 14.8 ± 8.1 mM (p = 0.00067) (Fig. 2B). During the incremental trial, the increases in sweat chloride concentration from 125–150 W (p = 0.003), 150–175 W (p = 0.0013), and 175–200 W (p = 0.007) were statistically significant (Fig. 2C**)**.

The changes in sweat chloride ion concentration (ΔC) in the 100–200 W and 200–100 W trials were correlated to the absolute value in the first segment (Fig. 2D,E): in general, a higher concentration in the 100 W segment resulted in a higher change on increasing the power output to 200 W. Similarly, a higher concentration in the 200 W segment resulted in a larger decrease on reducing the power output to 100 W. The p-values for the linear regression fits for each trial (Fig. 2D,E) were 0.051 and 0.024, respectively, and the Pearson correlation coefficients were 0.574 and −0.643, respectively. During the multi-step trial, the average concentration increase (ΔC) per 25 W was 6.2 ± 0.7 mM for exercise loads after the 125 W segment (Fig. 2F).

Comparison of sweat chloride concentrations at 200 W in the single step and reverse step trials shows that values were about the same for many of the subjects, although there was more than a two-fold difference for two subjects (Fig. 2G). The mean sweat chloride concentrations for all subjects at 100 W and 200 W were independent of the protocol (Fig. 2H). As described above, the sweat chloride concentrations at 200 W for all three protocols were statistically significantly higher than at 100 W.

**Figure 3:**
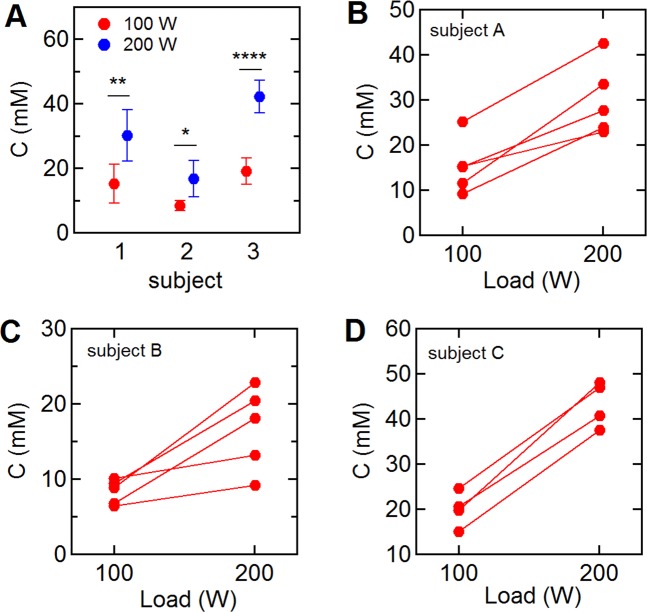
Intra-individual variation of sweat profiles for single step trials (100–200 W). Three subjects repeated the single step trial (100–200 W) five times on different days. (**A**) Average sweat chloride concentration at 100 and 200 W during the trials (red: 100 W, blue: 200 W). (**B**–**D**) Intra-individual variations in the sweat chloride concentration of subjects A, B, and C. Note that the 2nd and 5th trials for subject C were almost identical (2nd: 100 W = 15.0 mM, 200 W = 37.4 mM, 5th: 100 W = 15.0 mM, 200 W = 37.5 mM) and are hence not distinguishable on the plot. *p < 0.05, **p < 0.01, ***p < 0.001, ****p < 0.0001.

To assess intra-individual variations, three subjects repeated the single step (100 W–200 W) trial five times on different days. All repeat trials showed the same features, with an increase in sweat chloride on increasing the power output from 100 W to 200 W (Table S1). The sweat chloride concentration at each exercise load (100 W or 200 W) over the five trials had a standard deviation of 2–8 mM (subject A: 15.2 ± 6.1 mM (mean ± SD) at 100 W, 30.1 ± 8.1 mM at 200 W, subject B: 8.3 ± 1.6 mM, 16.7 ± 5.5 mM, subject C: 19.0 ± 4.0 mM, 42.1 ± 5.1 mM) (Fig. 3 and Table S2).

**Figure 4:**
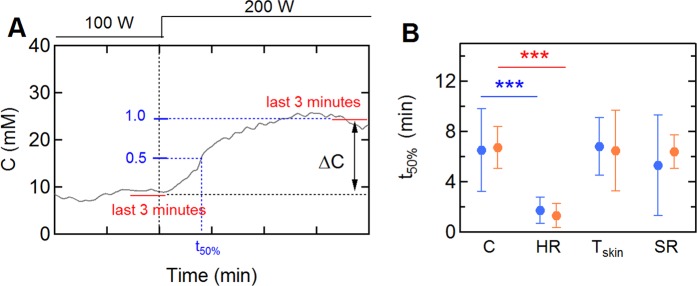
The transient response of sweat profiles and other physiological vitals on step changes in exercise load. (**A**) The half-time in sweat chloride concentration following a step change in exercise load. The value of ΔC is the difference between the average values for the last 3 minutes at each exercise load. (**B**) Average half-times of the sweat profiles (N = 11), heart rate (N = 12), skin temperature (N = 12), and SR (N = 12) for single step (100–200 W) and reverse step (200–100 W) trials. Blue: 100–200 W; red: 200–100 W. One sweat profile for which ΔC was <5 mM was excluded from analysis. ***p < 0.001.

The transient responses in the sweat profiles during the single step and reverse step trials were analyzed and the results compared with other vitals. We define the half-time (t_50%_) of the sweat chloride response to a step change in power output as the time required to reach 50% of the concentration change (ΔC) (Fig. 4A). A similar approach was used for the other vital signs. The half-times in sweat chloride during the single step trials (100–200 W) were 6.5 ± 3.3 min. During the reverse step trials (200–100 W), the values were slightly longer (t_50%_ = 6.7 ± 1.6 min) although the differences were not statistically significant. Comparison of the transient response in sweat chloride with other vital signs show similar half-times for skin temperature, and sweat rate, however, the changes in heart rate were significantly faster (Fig. 4B). Comparison of sensor results to laboratory measurement of samples collected during the trials highlights the challenges in assessing dynamic changes in real time (Fig. S1 in *Supplementary Information*).

### Changes in sweat concentration and sweat rate

**Figure 5:**
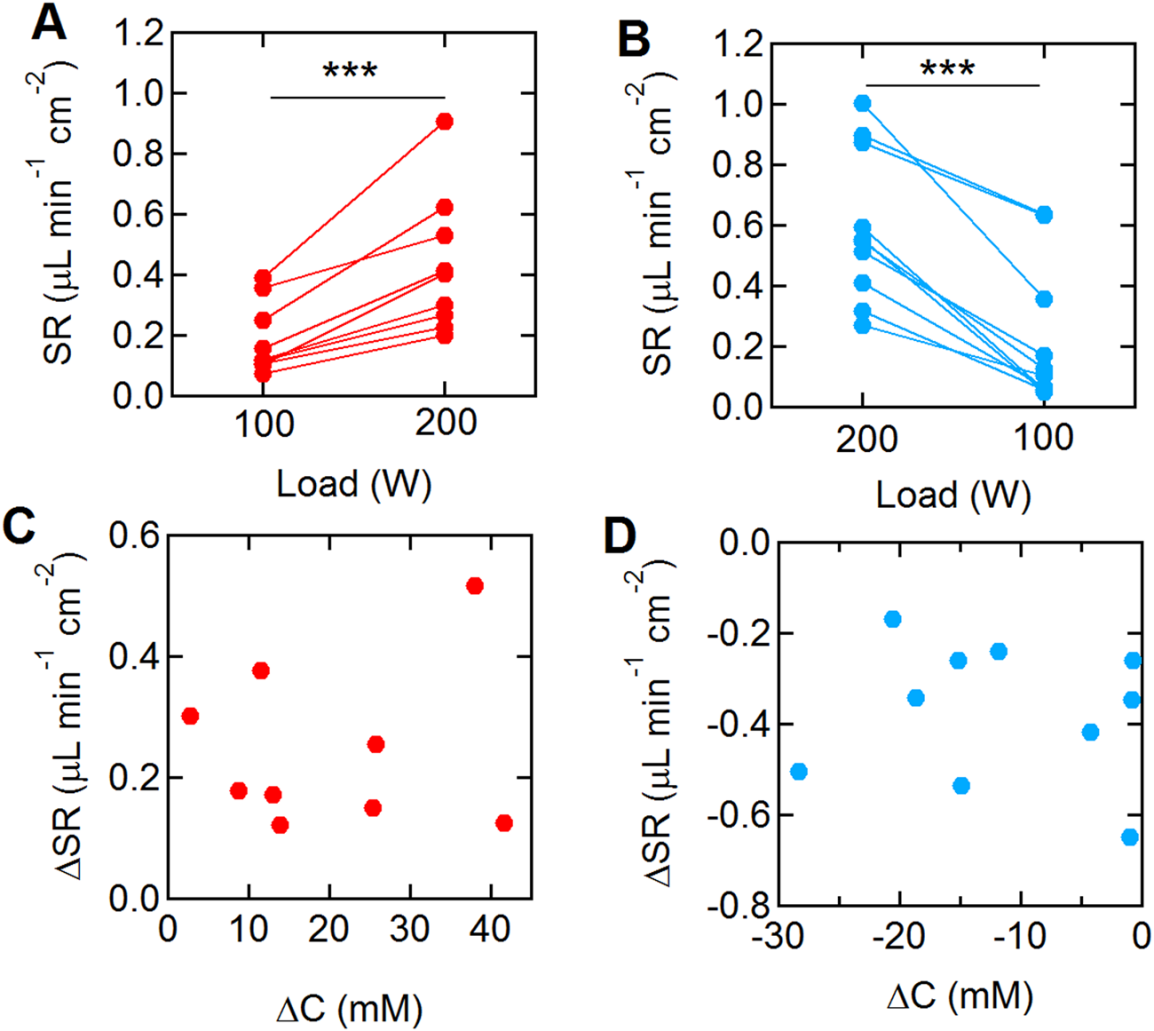
Changes in sweat rate at different exercise loads. (**A**) Sweat rate at 100 and 200 W during the single step trials (N = 9). (**B**) Sweat rate at 200 and 100 W during the reverse step trials (N = 10). (**C,D**) The correlation between changes in the sweat concentration (∆C) and sweat rate (∆SR) during (**C**) the single and (**D**) reverse step trials. N = 12. QNS and LOD samples were excluded. ***p < 0.001.

The sweat rate during each trial was obtained from the volume changes in the sweat sample collected in the Macroduct device. Here we consider the sweat rate over the last 2 minutes of each segment (100 W or 200 W). The sweat rate for all 12 subjects varied with exercise intensity (Fig. 5A,B). The average sweat rates (SR) at 100 and 200 W during the single step (100 W–200 W) trials were 0.19 ± 0.12 and 0.43 ± 0.23 μL min^−1^ cm^−2^, respectively (p < 0.001). During the reverse step trial (200 W–100 W), the sweat rates at 200 and 100 W were 0.60 ± 0.25 and 0.23 ± 0.3 μL min^−1^ cm^−2^, respectively (p < 0.001). The average changes in the sweat rate (ΔSR) were 0.24 ± 0.13 μL min^−1^ cm^−2^ (100 W–200 W) and −0.37 ± 0.15 μL min^−1^ cm^−2^ (200 W–100 W). Although the sweat chloride concentration and sweat rate for each individual changed in response to exercise load (Fig. S2 in *Supplementary Information*), there was no correlation between them (Fig. 5C,D). The Pearson correlation coefficients between ΔC and ΔSR during the single and the reverse step trials were 0.125 (p = 0.748) and −0.115 (p = 0.752), respectively. Furthermore, there was no correlation between overall weight loss and the sweat volume obtained from the forearm (Fig. S3 in *Supplementary Information*).

### Changes in sweat concentration and other physiological responses

Next we compared variations in sweat chloride concentration (ΔC) with other physiological parameters including heart rate (HR), skin and core temperature, and RPE, along with other participant information, including age, body mass index (BMI) and exercise frequency per week (f_ex_). To quantitively assess each subject’s fitness level, eight subjects performed PWC_170_ tests. PWC_170_ is a sub-maximal test used to estimate an individual’s aerobic fitness level^27,28^. However, there were no significant correlations between ΔC and physiological parameters or participant information (Table 1).

**Table 1:** The relation between changes in sweat chloride ion concentration (ΔC) and physiological parameters on increasing exercise load from 100 to 200 W (N = 12). ΔHR, ΔT_core_, ΔT_skin_, and ΔRPE were determined from ΔX = X_2_ – X_1_, where X_1_ and X_2_ are the average value for last 1 minute during 100 and 200 W segments, respectively.

Subject	∆C (mM)	PWC_170 _(bpm)	age (years)	BMI	f_ex_	HR_max_ (bpm)	∆HR (bpm)	∆T_core_ (˚C)	∆T_skin_ (°C)	∆RPE
1	41.6	197.2	18.0	22.7	6.0	202.0	33.7	1.1	0.6	4.0
2	25.8	159.9	32.0	22.6	5.0	188.0	47.5	1.1	0.6	5.1
3	11.5	144.5	25.0	22.2	7.0	195.0	53.5	1.6	1.1	6.0
4	25.4	301.6	25.0	23.4	7.0	195.0	57.9	0.7	0.3	1.9
5	25.7	321.7	28.0	23.1	4.5	192.0	46.6	1.5	0.7	1.9
6	8.7	225.6	29.0	24.8	3.5	191.0	43.0	0.9	1.1	5.0
7	2.7	N/A	21.0	24.5	4.0	199.0	46.3	1.2	0.4	3.1
8	12.3	N/A	26.0	24.4	6.0	194.0	35.3	2.7	0.0	4.0
9	38.0	192.8	20.0	26.7	4.5	200.0	55.1	1.0	0.2	3.9
10	13.9	N/A	33.0	27.9	6.0	187.0	46.0	1.1	−0.1	3.9
11	13.0	N/A	24.0	23.5	6.0	196.0	42.1	1.2	0.6	5.0
12	13.8	216.8	19.0	23.4	7.0	201.0	43.7	0.6	0.5	4.0
correlation with ∆C	1.00	0.05	−0.27	−0.08	0.05	0.27	0.07	−0.23	−0.15	−0.24

### Comparison of sweat chloride and sweat rate during exercise and following chemically-induced sweat induction

To compare the sweat chloride and sweat rate responses between exercise-induced sweating and chemically-induced sweating, we performed conventional pilocarpine iontophoresis laboratory tests on 8 subjects who participated the single step (100–200 W) trial. The sweat chloride profiles for chemically-induced sweat were generally flat with a constant value during the 30 minute test. From analysis of the sweat collection device, the sweat rate for chemically-induced was initially high, typically 0.4–0.8 μL min^−1^ cm^−2^, and decreased approximately linearly over the measurement period (Fig. S4 in *Supplementary Information*).

**Figure 6:**
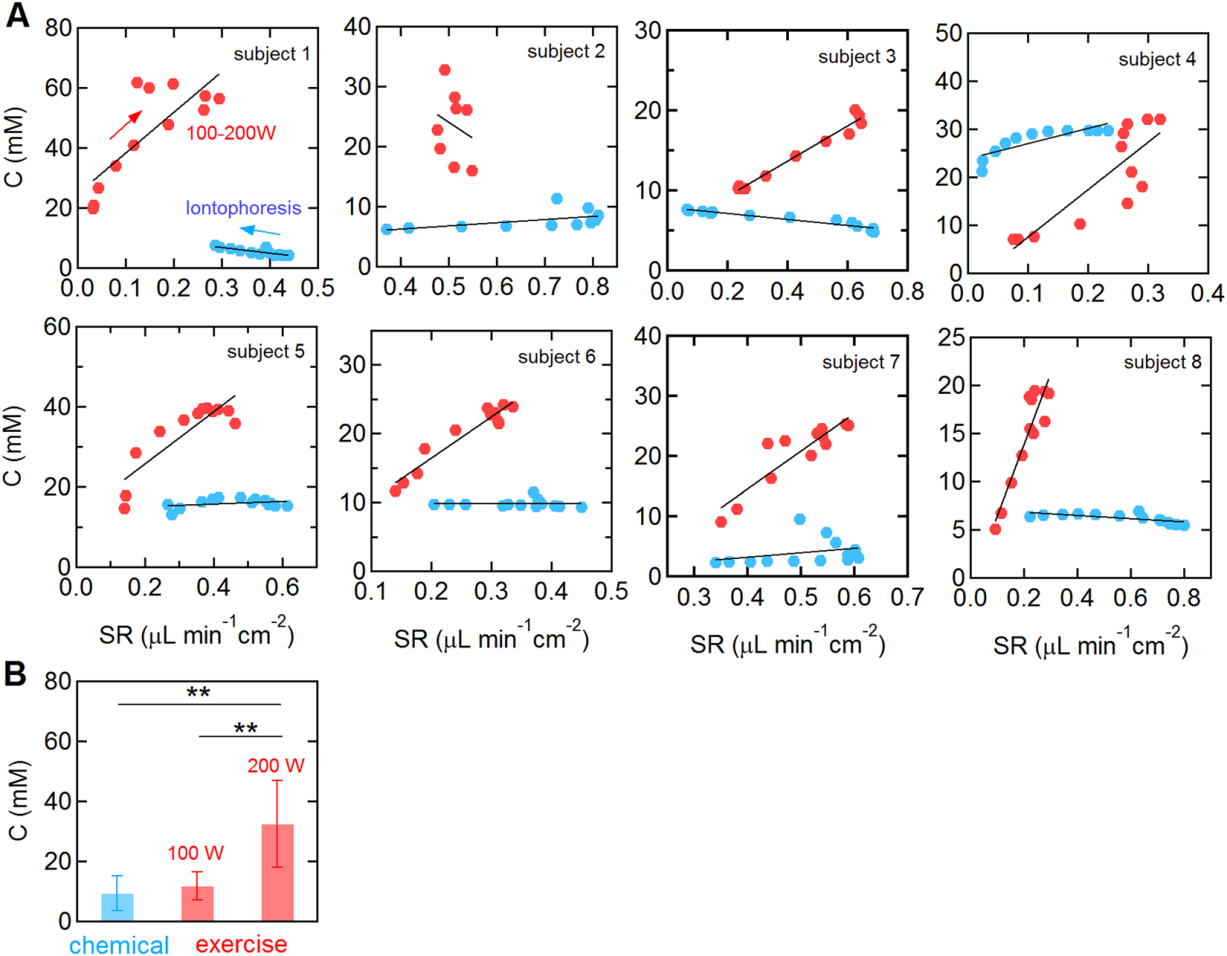
Comparison of chemically-induced and exercise-induced sweating. (**A**) Sweat chloride versus sweat rate during exercise (100 W–200 W trial) and following chemical sweat induction for 8 subjects. The concentration values at each point were obtained by averaging the sweat profile measured every 2 minutes (red: exercise, blue: pilocarpine iontophoresis, solid line: linear regression). For exercise trials, the sweat concentration and sweat rates were analyzed during the 20-minute 200 W segment. Following chemical sweat induction, data were collected as soon a stable sweat chloride concentration was detected (typically 2–3 minutes following attachment of the sensor). (B) Average sweat chloride concentration in response to chemically-induced sweating and exercise-induced sweating at 100 and 200 W (N = 8). The sweat concentration following chemically-induced sweating was obtained by averaging the sensor output for the last 2 minutes of the 30 minute measurement. **p < 0.01.

For chemically-induced sweating, the sweat chloride concentration was only weakly dependent on sweat rate (Fig. 6). The average slope and y-intercept were 2.3 ± 14.3 mM min cm^2^ μL^−1^ and 10.3 ± 7.2 mM, respectively (subject 1,3,4, and 8: p < 0.05, the others: p > 0.05). In contrast, for exercise-induced sweating, the sweat chloride concentration was strongly dependent on sweat rate. From linear regression, the average slope and y-intercept were 55.5 ± 53.9 mM min cm^2^ μL^−1^ and 11.0 ± 19.1 mM, respectively (p < 0.01, except subject 2). In addition, the sweat chloride concentration was typically higher during exercise than following chemical sweat induction. The average sweat chloride concentration for the eight subjects was 9.3 ± 5.7 mM following chemically-induced sweating. The sweat chloride concentrations at 100 W were slightly higher (11.7 ± 4.7 mM) but not statistically significant (p = 0.43). However, the concentration at 200 W (32.4 ± 14.5 mM, p = 0.003) was statistically significantly higher than for chemically-induced sweating.

## Discussion

In response to step changes in exercise intensity we observed corresponding changes in sweat chloride concentration (Fig. 2). The average change in sweat chloride on increasing the exercise load from 100 W to 200 W was about 20 mM, which was reversible on decreasing the load. These results suggest that exercise load is an important factor in assessing electrolyte loss, especially for endurance athletes. Since electrolyte loss is related to the product of sweat chloride (or sodium) concentration and sweat rate, an increase in sweat chloride concentration at higher loads where the sweat rate is also increased, amplifies the effect.

The sweat chloride concentration is generally thought to increase with increasing sweat rate due to an associated decrease in the reabsorption efficiency in the sweat ducts^2,23,24,29^, which is thought to be dependent on factors such as fitness level, training status, and heat acclimation^29,30^. An approximately linear dependence has been reported for exercise-induced sweating^23,24^. Here we also show a linear relationship between sweat chloride and sweat rate for 7 of 8 subjects in the 200 W segment of the single step trial (Fig. 6). However, we found no correlation between the average values of ΔC and ΔSR, fitness level, or other physiological parameters (Fig. 5 and Table 1). The lack of correlation between ∆C and ΔSR or other parameters could be due to the magnitude of intra-individual variations (Fig. 3) or differences in heat acclimation.

The coefficients of variation in sweat chloride concentration for individuals performing repeat single step trials were 12 to 40%. Previous studies of sweat chloride following repeated pilocarpine iontophoresis tests (8 repeated tests on 12 individuals) reported values of intra-individual variation of from 9.7% to 45.2%^31^, similar to the values found here during exercise trials. These results imply that the origin of the intra-individual variation may be the same for both exercise-induced and chemically-induced induction.

The half-times for the sweat chloride response to a step change in exercise load were 3–10 minutes. As we show below, the half-times associated with sweat transit in the ducts, and the response time of the sensor are much faster, therefore, we can conclude that the half-times for the sweat chloride are coupled to an increase sweat rate and a decrease in absorption efficiency. In a study of passive heating, the sweat rate was found to be proportional to skin temperature^32^, implying that the half-time should be similar to values for sweat rate and sweat chloride concentration, as observed here.

For a typical sweat gland that is 2 mm long and 15 µm in diameter, the transit time in a duct for sweat rates of 0.001–0.005 µL min^−1^ gland^−1^ (0.1–0.5 µL min^−1^ cm^−2^) would be 4–22 seconds. The response time of the sensor is associated with the time to replace the sweat volume between the sensor and the skin. Assuming that skin has 40 µm deep parallel grooves with a line density of 40 cm^−1^ ^33^, the sweat volume under the sensor is 0.37 µL cm^−2^. For sweat rates of 0.1–0.5 µL min^−1^ cm^−2^, the time to replace the volume under the sensor would be 0.75–3.7 minutes.

The responses to exercise- and chemically-induced sweating were significantly different. In chemically-induced sweating the sweat rate decreased with time although the sweat chloride concentration remained almost constant (Fig. 6). In contrast, during exercise, the sweat rate increased with exercise intensity and resulted in a significant increase in sweat chloride concentration (Figs. 1, 2). Furthermore, at the same sweat rate, the sweat chloride concentration was significantly higher during exercise compared to chemically-induced sweating for most of the subjects (Fig. 6).

The difference between the two induction methods could be due to differences in the open probability of the cystic fibrosis transmembrane conductance regulator (CFTR) ion channel. The CFTR channel is an ATP-gated anion channel which facilitates reabsorption of chloride ions in the sweat duct^34–37^. Since the sweat chloride concentration is approximately constant in response to chemically-induced sweating, at least for sweat rates lower than about 0.8 μL min^−1^ cm^−2^, we assume that the open probability remains constant. However, electrophysiology studies have shown that the channel open probability is dependent on ATP concentration^37^ and hence nutrient depletion in ductal epithelial cells during exercise (e.g. from insufficient oxygen for oxidative phosphorylation) could decrease the channel open probability. Therefore, we speculate that an increase in exercise intensity could decrease the channel open probability and hence reduce reabsorption efficiency and increase the sweat chloride concentration.

In summary, we show that wearable sensors enable real-time measurements of the dynamic response to step changes in exercise load. Real-time measurements allow direct assessment of transient changes in sweat chloride concentration, with a response time similar to the transit time of an ion in a sweat duct at a moderately fast sweat rate. In addition, real-time measurements do not require subsequent laboratory analysis and require a smaller volume for measurement. On changing the exercise load from 100 to 200 W or 200 W to 100 W we observed statistically significant changes in sweat chloride concentration with half-times on the order of 6 minutes. However, there was no correlation between changes in sweat chloride concentration and changes in sweat rate or other physiological parameters. We also show that individuals show significantly different responses to exercise- and chemically-induced sweating.

## Methods and Materials

### Sweat sensor

A wearable potentiometric sweat sensor with an integrated salt bridge was employed to measure the sweat chloride concentration in real time (Fig. S5 in *Supplementary Information*)^11,15,38^. The output voltage of the sensor is dependent on the chloride concentration in sweat^11^. Prior to each trial, the sensor was calibrated with sodium chloride solutions of 10, 50, and 100 mM at 21 °C. Sweat chloride concentrations were corrected to 32 °C, a typical skin temperature, using the Nernst equation.

### On-body exercise trials

All on-body trials were performed under a protocol approved by the institutional review board (IRB) at Johns Hopkins University (IRB00122647). All experiments were performed in accordance with guidelines and regulations. All subjects read the study information document and provided written informed consent before participation. In addition, participants completed a health screening questionnaire to ensure the absence of thermoregulatory and cardiovascular disease, or other conditions that may alter their physiological response, sweat rate, or electrolyte concentration.

12 healthy individuals (9 males, 3 females) participated in this study to assess dynamic changes in sweat concentration in response to changes in exercise load. The mean age of the subjects was 25.3 ± 4.8 (mean ± SD) and the mean body mass index (BMI) was 24.1 ± 1.7. Participants reported that they worked out 5.6 ± 1.2 days per a week. The subjects fasted for at least three hours before the start of the trial. Subjects were provided water before each trial (5 mL kg^−1^ of body weight) and did not eat or drink during the trial. Identical t-shirts and shorts were provided to all subjects. The subjects were asked to spin on a stationary bike (Keiser, M3i) following three different exercise protocols after a warm up (55 W for 15 minutes): (1) a step trial: 100 W (20 minutes) to 200 W (20 minutes), (2) reverse step trial: 200 W (20 minutes) to 100 W (10 minutes), and (3) multi-step trial: 100 W – 200 W in 25 W increments for 10 minutes each. The three trials for each subject were performed on three different days with a minimum interval between the trials of 24 hours. The order of the three trials was randomized. All trials were completed between February and November 2019. Room temperature and humidity were maintained at 24.8 ± 0.2 °C and 41.8 ± 15.5%, respectively. Trial preparation and warm up were performed in the same room under the same conditions. The stationary bike was calibrated regularly following the manufacturer’s instructions.

To assess variations in sweat profiles, three subjects (3 males, age = 22.3 ± 5.8 years, BMI = 23.3 ± 1.4, exercise frequency a week = 5.2 ± 1.4 days week,) repeated the step trial (100–200 W) five times on different days. Two of the three subjects also participated in the three exercise protocol trials described above. The repeated trials were completed within 196, 50, 49 days, for subjects subject A, B and C, respectively (Table S1 in *Supplementary Information*).

### Data collection and analysis

During the trial, the sweat chloride concentration (C), sweat rate (SR), heart rate (HR), and skin temperature (T_skin_) were measured using wearable sensors (Fig. S2 in *Supplementary Information*). The sweat chloride sensor was attached to the left ventral forearm using a commercial bandage (Tegarderm, 3 M) and a wrist band (Under Amour), and its output was monitored via a custom-made Bluetooth transceiver and smartphone app^15^. Prior to each trial, the subject’s forearm was cleaned using 70% isopropyl alcohol and deionized water. A bio-harness (Zephyr, Medtronics) and a thermocouple sensor (Neulog, Fisher) were used to measure the heart rate and the skin temperature, respectively. The thermocouple was attached on the right forearm by a commercial bandage (Tegarderm, 3 M) and covered by a wrist band (Under Amour). RPE and tympanic temperature values were recorded every 5 minutes and linear interpolation was used to obtain data points every second. Tympanic temperature was used as an estimate of core temperature (T_core_). Two Macroduct sweat collection devices (Wescor) were attached to the right ventral forearm. One was used to collect sweat samples for laboratory analysis and the other was used to determine the sweat rate. Two sweat samples were collected during each trial by changing the Macroduct device used to determine sweat chloride concentration at the step change in output power (single step: at the increase in exercise load from 100 to 200 W; reverse step: at the decrease from 200 to 100 W; multi-step: at the increase from 150 to 175 W). The collected sweat samples were analyzed in the clinical chemistry laboratory at Johns Hopkins Hospital using coulometric titration (Wescor, Chlorocheck) following the Cystic Fibrosis Foundation guidelines^14^.

At the beginning of the trial, the initial transient response was due to the high sensor impedance caused by insufficient sweat and does not reflect a high sweat chloride concentration. On generation of sufficient sweat for equilibration the sensor output quickly decreased to a steady state value (Fig. 1). To establish a steady state response, we define the sweat chloride concentration in the first plateau region where the slope of the sweat profile is smaller than 0.1 mM min^−1^ for at least 5 minutes. Plateau regions were determined using a sequential linear regression (least squares fit) in a moving 5-minute window using a customized MATLAB code^30^. The start times of the plateau region for 12 subjects during the single and reverse step trials were 21.1 ± 7.3 and 18.0 ± 4.4 minutes, respectively. Since the exercise load was changed at 35 minutes after the start of the trial, the sensor stabilization time associated with the onset of sweating is well before the dynamic changes in sweat chloride concentration. For the step trials, the sweat concentration for three subjects did not reach a plateau in the first 100 W segment (Fig. 2C). However, at 125 W all sweat concentrations satisfied the criteria for a plateau. For all analysis reported here we obtained  the average sweat chloride concentration over the last 1 minute at each exercise load.

The sweat rate was determined from images of the Macroduct coil taken every 2 minutes, using a previously validated LabVIEW algorithm^15^. SPSS (IBM) was employed for statistical analysis and a customized MATLAB code was used for signal filtering, data interpolation, and analysis of the transient response. To reduce high frequency noise in the sensor output, a median filter with a 1-minute window was applied to the voltage versus time curves.

### Pilocarpine iontophoresis

To compare exercise-induced sweating to chemically-induced sweating, we recorded the sweat chloride concentration and sweat rate in response to pilocarpine iontophoresis. Iontophoresis was performed sequentially on both arms (Wescor, Sweat Inducer 3700). A Macroduct (Wescor) was attached to one arm and images of the Macroduct taken every 2 minutes to measure the sweat rate. A sweat chloride sensor was attached on the other arm using an adhesive bandage (Tegarderm, 3 M) and its output was monitored in real time using the smartphone app. The sweat tests using pilocarpine iontophoresis were performed on different days from the exercise trials.

### PWC170 (Physical working capacity at a heart rate of 170 bpm) test

The PWC_170_ test has been widely used to quantify fitness level^39,40^. PWC_170_ was determined for 8 subjects (5 male and 3 female). The test consisted of three steps while spinning at 60 rpm. The first step was 3 minutes long at an exercise load of about 50 Watts, with the subject’s heart rate below 120 bpm. The second step was 6 minutes long, with the exercise load adjusted over the first 3 minutes until the heart rate was between 110–130 bpm, and then continued for an additional 3 minutes. The third step was 6 minutes long, with the exercise load further increased for the first 3 minutes until the heart rate was between 140–160 bpm, and then continued for an additional three minutes. For all steps, if the heart rate reached or exceeded 170 bpm, the test was terminated. The average heart rate and power output were obtained from the final minute of the second and third steps and used to extrapolate the power output at a heart rate of 170 bpm from a linear fit. The predicted power output (W) at 170 bpm (PWC_170_) was calculated from the extrapolation.

## Supplementary information

Supplementary Information.

## Data Availability

The data that support the findings of this study are available from the corresponding author upon request.

## References

[1] Hu Y, Converse C, Lyons MC, Hsu WH (2018). Neural control of sweat secretion: a review. Br. J. Dermatol..

[2] Quinton PM (1983). Sweating and Its Disorders. Annu. Rev. Med..

[3] Shibasaki M, Crandall CG (2010). Mechanisms and controllers of eccrine sweating in humans. Front. Biosci..

[4] Baker LB (2019). Physiology of sweat gland function: The roles of sweating and sweat composition in human health. Temperature.

[5] Boysen TC, Yanagawa S, Sato F, Sato K (1984). A modified anaerobic method of sweat collection. J. Appl. Physiol. Respir. Env. Exerc. Physiol.

[6] Buono MJ, Lee NVL, Miller PW (2010). The relationship between exercise intensity and the sweat lactate excretion rate. J. Physiol. Sci..

[7] Dziedzic CE, Ross ML, Slater GJ, Burke LM (2014). Variability of Measurements of Sweat Sodium Using the Regional Absorbent-Patch Method. Int. J. Sport. Physiol..

[8] Baker LB (2017). Sweating Rate and Sweat Sodium Concentration in Athletes: A Review of Methodology and Intra/Interindividual Variability. Sports Med..

[9] Gao W (2016). Fully integrated wearable sensor arrays for multiplexed *in situ* perspiration analysis. Nature.

[10] Bandodkar AJ (2014). Epidermal tattoo potentiometric sodium sensors with wireless signal transduction for continuous non-invasive sweat monitoring. Biosens. Bioelectron..

[11] Choi DH, Li Y, Cutting GR, Searson PC (2017). A wearable potentiometric sensor with integrated salt bridge for sweat chloride measurement. Sens. Actuat B-Chem.

[12] Guinovart T, Bandodkar AJ, Windmiller JR, Andrade FJ, Wang J (2013). A potentiometric tattoo sensor for monitoring ammonium in sweat. Analyst.

[13] Rose DP (2015). Adhesive RFID Sensor Patch for Monitoring of Sweat Electrolytes. IEEE Trans. Biomed. Eng..

[14] LeGrys VA (2007). Diagnostic sweat testing: the Cystic Fibrosis Foundation guidelines. J. Pediatr..

[15] Choi DH (2018). Sweat test for cystic fibrosis: Wearable sweat sensor vs. standard laboratory test. J. Cyst. Fibros..

[16] Berend K, van Hulsteijn LH, Gans RO (2012). Chloride: the queen of electrolytes?. Eur. J. Intern. Med..

[17] Lewis, D., Blow, A., Tye, J. & Hew-Butler, T. Considering exercise-associated hyponatraemia as a continuum. *BMJ Case Rep* 2018, 10.1136/bcr-2017-222916 (2018). 10.1136/bcr-2017-222916 PMC5847945 29523608

[18] Adrogue HJ, Madias NE (2000). Hyponatremia. N. Engl. J. Med..

[19] Augarten A (1995). The significance of sweat Cl/Na ratio in patients with borderline sweat test. Pediatr. Pulmonol..

[20] Bijman J (1984). & Quinton, P. M. Influence of abnormal Cl- impermeability on sweating in cystic fibrosis. Am. J. Physiol..

[21] Emrich HM (1968). Sweat composition in relation to rate of sweating in patients with cystic fibrosis of the pancreas. Pediatr. Res..

[22] Sonner Z (2015). The microfluidics of the eccrine sweat gland, including biomarker partitioning, transport, and biosensing implications. Biomicrofluidics.

[23] Buono MJ, Ball KD, Kolkhorst FW (2007). Sodium ion concentration vs. sweat rate relationship in humans. J. Appl. Physiol..

[24] Buono MJ, Claros R, DeBoer T, Wong J (2008). Na+ secretion rate increases proportionally more than the Na+ reabsorption rate with increases in sweat rate. J. Appl. Physiol..

[25] Dill DB, Hall FG, Van Beaumont W (1966). Sweat chloride concentration: sweat rate, metabolic rate, skin temperature, and age. J. Appl. Physiol..

[26] Shamsuddin AK (2005). Changes in the index of sweat ion concentration with increasing sweat during passive heat stress in humans. Eur. J. Appl. Physiol..

[27] Heyman E, Briard D, Dekerdanet M, Gratas-Delamarche A, Delamarche P (2006). Accuracy of physical working capacity 170 to estimate aerobic fitness in prepubertal diabetic boys and in 2 insulin dose conditions. J. Sports Med. Phys. Fit..

[28] Rowland TW, Rambusch JM, Staab JS, Unnithan VB, Siconolfi SF (1993). Accuracy of physical working capacity (PWC170) in estimating aerobic fitness in children. J. Sports Med. Phys. Fit..

[29] Araki T, Matsushita K, Umeno K, Tsujino A, Toda Y (1981). Effect of physical training on exercise-induced sweating in women. J. Appl. Physiol. Respir. Env. Exerc. Physiol.

[30] Choi DH (2019). Two Distinct Types of Sweat Profile in Healthy Subjects While Exercising at Constant Power Output Measured by a Wearable Sweat Sensor. Sci. Rep..

[31] Willems P, Weekx S, Meskal A, Schouwers S (2017). Biological Variation of Chloride and Sodium in Sweat Obtained by Pilocarpine Iontophoresis in Adults: How Sure are You About Sweat Test Results?. Lung.

[32] Nadel, E. R., Bullard, R. W. & Stolwijk, J. A. Importance of Skin Temperature in Regulation of Sweating. *J Appl Physiol* **31**, 80-& (1971). 10.1152/jappl.1971.31.1.80 5556967

[33] Dussaud AD, Adler PM, Lips A (2003). Liquid transport in the networked microchannels of the skin surface. Langmuir.

[34] Hwang TC, Kirk KL (2013). The CFTR ion channel: gating, regulation, and anion permeation. Cold Spring Harb. Perspect. Med..

[35] Cai Z, Taddei A, Sheppard DN (2006). Differential sensitivity of the cystic fibrosis (CF)-associated mutants G551D and G1349D to potentiators of the cystic fibrosis transmembrane conductance regulator (CFTR) Cl- channel. J. Biol. Chem..

[36] Ishihara H, Welsh MJ (1997). Block by MOPS reveals a conformation change in the CFTR pore produced by ATP hydrolysis. Am. J. Physiol..

[37] Sheppard DN, Welsh MJ (1999). Structure and function of the CFTR chloride channel. Physiol. Rev..

[38] Choi DH, Kim JS, Cutting GR, Searson PC (2016). Wearable Potentiometric Chloride Sweat Sensor: The Critical Role of the Salt Bridge. Anal. Chem..

[39] Beltz NM (2016). Graded Exercise Testing Protocols for the Determination of VO2max: Historical Perspectives, Progress, and Future Considerations. J. Sports Med..

[40] Jonsson BG, Astrand I (1979). Physical work capacity in men and women aged 18 to 65. Scand. J. Soc. Med..

