# Supplementary material for: The Dynamic Response of Sweat Chloride to Changes in Exercise Load Measured by a Wearable Sweat Sensor
Source: Sci Rep. 2020 May 7;10:7699. doi: 10.1038/s41598-020-64406-5 (PMC7205967; doi:10.1038/s41598-020-64406-5)
Supplement: Supplementary file 1 — Supplementary Information. [file 41598_2020_64406_MOESM1_ESM.pdf]

Supplementary Information

The Dynamic Response of Sweat Chloride to Changes in Exercise Load Measured by a Wearable Sweat Sensor

Dong-Hoon Choi[1], Grant Kitchen[1], Kerry J. Stewart[2], and Peter C. Searson[*1,3]

[1] Institute for Nanobiotechnology, Johns Hopkins University, Baltimore, USA

[2] Department of Medicine, Johns Hopkins University School of Medicine, Baltimore, USA

[3] Department of Materials Science and Engineering, Johns Hopkins University, Baltimore, USA

[*] Corresponding author

E-mail: searson@jhu.edu

Table S1. Sweat chloride concentrations (mM) for three subjects (A, B, and C) during repeated single step trials (100 – 200 W).

Table S2. Summary of sweat chloride concentration (mM) and coefficient of variation (CV) for three subjects (A, B, and C) during repeated single step trials (100 – 200 W). **Table S3.** Summary of sweat chloride concentration for individuals during single step (100 – 200 W) trials and reverse step (200 - 100 W) trials.

Table S3. Mean and standard deviation of sweat chloride concentrations, along with the coefficient of variation, for individuals during single step (100 – 200 W) and reverse step (200 - 100 W) trials.

Figure S1. Wearable sweat sensor versus a conventional laboratory-based sweat test.

Figure S2. Typical profiles of sweat chloride concentration, heart rate, the skin temperature, and local sweat rate for one subject during single step, reverse step, and multistep trials.

Figure S3. Whole body sweat loss and forearm sweat loss.

Figure S4. Sweat rate for 8 healthy individuals over time following pilocarpine iontophoresis.

Figure S5. Wearable sweat chloride sensor.

Intra-individual variation in sweat chloride concentration

Three subjects (A, B, and C) repeated the single step trials (100 – 200 W) five times on different days.

Table S1. Sweat chloride concentrations (mM) for three subjects (A, B, and C) during repeated single step trials (100 – 200 W).

	Subject A			Subject B			Subject C		
Trials	C_{100W}	C_{200W}	date	C_{100W}	C_{200W}	date	C_{100W}	C_{200W}	date
1	15.3	23.0	04/11	6.7	18.1	09/27	20.5	40.7	10/26
2	9.1	23.9	08/31	8.8	22.9	10/26	15.0	37.4	11/09
3	15.1	27.7	10/08	9.4	20.4	11/01	19.7	48.1	11/15
4	25.1	42.5	10/15	10.0	13.2	11/08	24.6	46.9	12/06
5	11.5	33.4	10/24	6.4	9.2	11/16	15.0	37.4	12/14

Table S2. Summary of sweat chloride concentration (mM) and coefficient of variation (CV) for three subjects (A, B, and C) during repeated single step trials (100 – 200 W). Coefficient of variation (CV) is the ratio of the standard deviation to the mean. The coefficient of variation (CV) for the repeat trials varied from 12.1 to 40.1 % which is slightly smaller than the CV among 12 individuals (37.9 to 54.7 %; see Table S3).

		Subject A	Subject B	Subject C
100 W (100-200W trial) N=5		15.2 ± 6.1 mM	8.3 ± 1.6 mM	19.0 ± 4.0 mM
200 W (100-200W trial) N=5		30.1 ± 8.1 mM	16.7 ± 5.5 mM	42.1 ± 5.1 mM
CV 100W	All Trials	40.1 %,	19.2 %,	21.1%
CV 200 W	All Trials	26.9 %	32.9 %	12.1 %

Table S3. Mean and standard deviation of sweat chloride concentrations, along with the coefficient of variation, for all individuals during single step (100 – 200 W) and reverse step (200 - 100 W) trials.

	**All individuals (N = 12)**
100 W (100-200W trial)	12.0 ± 5.9
200 W (100-200W trial)	31.4 ± 16
CV 100W (100-200W trial)	49.0 %
CV 200 W (100-200W trial)	51.0 %
200W (200-100W trial)	27.7 ± 10.5
100 W (200-100W trial)	14.8 ± 8.1
CV 200W (200-100W trial)	37.9 %
CV 100 W (200-100W trial)	54.7 %

Sweat sensor versus laboratory measurement

To compare the results from the sweat sensor to conventional laboratory measurements, sweat samples were collected using a Macroduct device attached to the right forearm for the single step and reverse step trials. Samples were obtained at each exercise intensity by changing the device at the step change from 100 W to 200 W or 200 W to 100 W. The collected sweat samples were analyzed by a conventional laboratory-based sweat test method.

The dynamic changes measured by the sensor (**Figs. 1- 4)** were not observed in the laboratory tests (**Fig. S1A-C**), which represent the average sweat chloride concentration over the collection period. A representative example (**Fig. S1A**) shows the limitations of laboratory-based tests. During the reverse step trial, the sweat concentration measured by the sensor increased up to 31 mM during 200 W segment, and then decreased to 16.7 mM during the subsequent 20 minutes at 100 W. The sweat chloride values for the laboratory tests (C_{lab}) were 20 mM for the sample collected during the 200 W segment, and 25 mM for the 100 W segment. The laboratory measurements (C_{lab}) for all samples collected in the single step trials were 18.6 ± 6.9 mM at 100 W and 36.0 ± 19.2 mM at 200 W (N = 5) (**Fig. S1B**). We note that 7 of 12 trials had at least one sample with a volume < 15 µL (QNS: quantity not sufficient) or a concentration < 10 mM (LOD: limit of detection). The average concentrations at 200 and 100 W during the reverse trials were 21.0 ± 8.2 and 24.3 ± 9.6 mM, respectively (N = 8; 4 trials were QNS or LOD) (**Fig. S1C**). Both results showed no statistical significance.

To compare the sweat chloride values obtained from the sensor and sweat samples (C_{lab}), we calculated the average sensor value (C_s) at each exercise load. The values were compared using a Bland-Altman plot (**Fig. S1D**), commonly used to assess the agreement between two quantitative measurements [1]. The mean difference ($C_s - C_{lab}$) was 0.3 mM and the standard deviation was 9.1 mM which is similar to the biological difference between left and right forearms. The mean difference of sweat concentrations obtained from left and right forearms using conventional laboratory-sweat tests was 0.5 mM and its standard deviation as 5.7 mM [2].

1. D. Giavarina. Understanding Bland Altman analysis. *Biochem Med (Zagreb)* 25, 141-51 (2015).
2. V. A. LeGrys, T. C. Moon, J. Laux, M. J. Rock and F. Accurso. Analytical and biological variation in repeated sweat chloride concentrations in clinical trials for CFTR modulator therapy. *J Cyst Fibros* 17, 43-49 (2018).

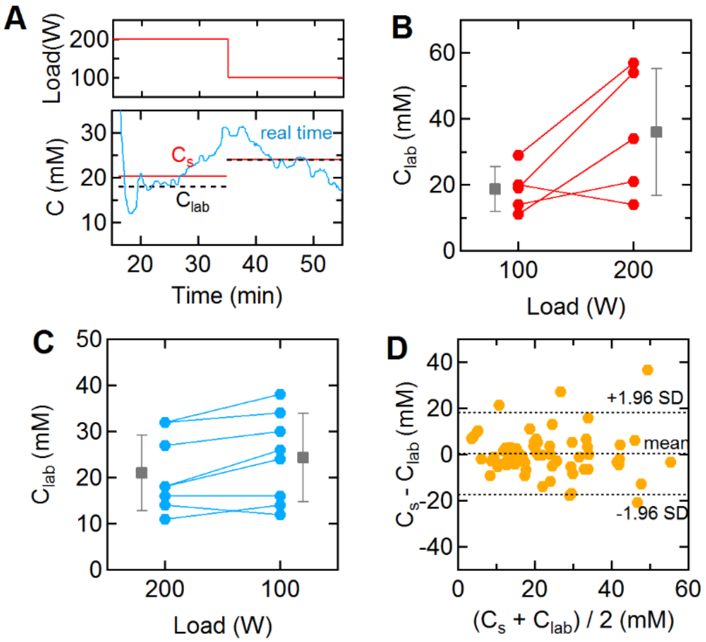

Figure S1. Wearable sweat sensor versus a conventional laboratory-based sweat test. (A) Sweat profile and laboratory test results. Solid red lines (C_s): average sensor output over the 100 W and 200 W segments. Dotted black line (C_{lab}): laboratory results from sweat samples collected during the 100 W and 200 W segments (using a Macroduct). Values were not obtained for sweat samples < 15 µL (QNS: quantity not sufficient) or concentrations < 10 mM (LOD: limits of detection) and hence QNS (N = 19) and LOD (N = 10) samples were excluded. The difference between average sensor values and laboratory results was close to zero, showing that there is no bias between the two measurements. (B, C) Laboratory results on sweat samples collected during (B) the single step (N = 5) and (C) reverse step trials (N = 8). QNS and LOD samples were excluded. (•) Mean ± SD at each exercise load. (D) Bland-Altman plot of the average sensor concentration (C_s) and laboratory test results (C_{lab}) from the Macroduct samples (N = 69).

Representative sweat profiles and other vitals

In this work, we measured sweat concentration (C), heart rate (HR), core (T_{core})and skin (T_{skin}) temperature and sweat rate at three different exercise protocol. **Fig. S2** shows typical dynamic changes of the sweat profile and the other vitals obtained from an identical participant at the three protocols. When the exercise load abruptly increased from 100 to 200 W, the sweat concentration started to increase and reached to higher concentration of 21.3 mM. The conventional laboratory sweat test results during 100 and 200 W were 14 and 21 mM, respectively (dotted line in **Fig. S2A**).

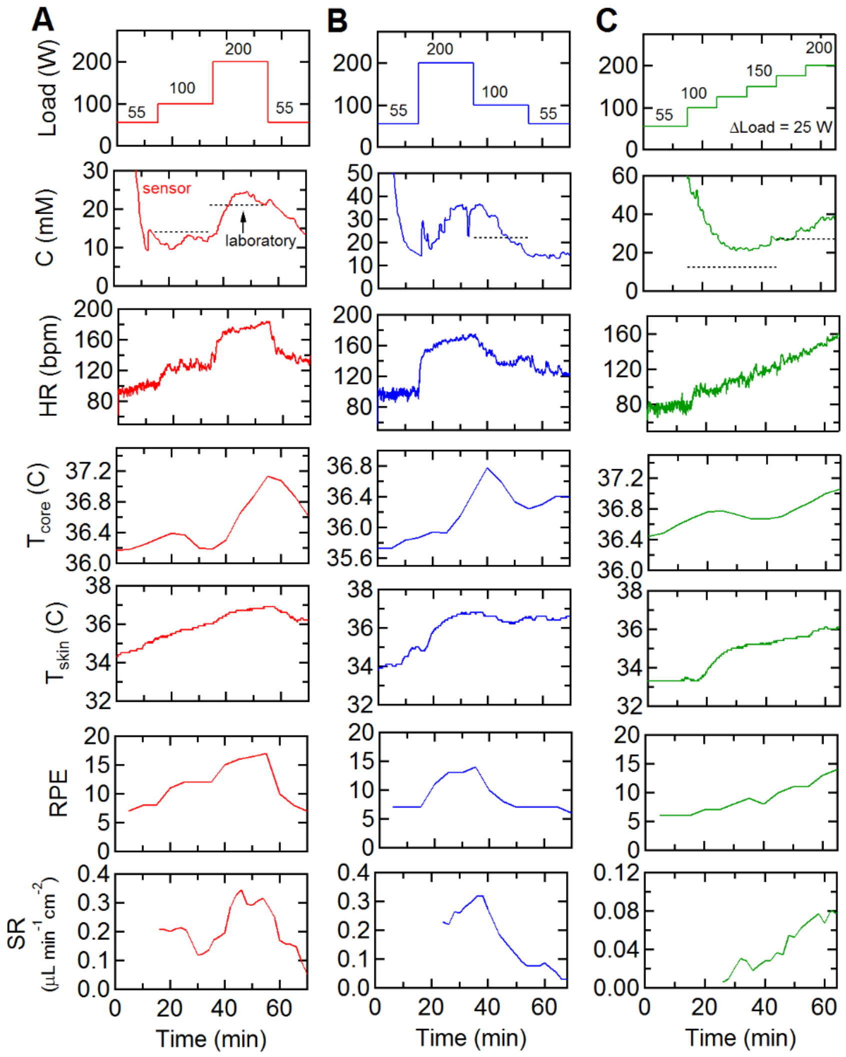

Figure S2. Typical profiles of sweat chloride concentration, heart rate, the skin temperature, and local sweat rate for one subject during single step, reverse step, and multistep trials. (A) Single step trial (100 W for 20 minutes, 200 W for 20 minutes), (B) reverse step trial (200 W for 20 minute, 100 W for 20 minutes), and (C) multi-step trial (100 – 125 – 150 – 175 – 200 W, each step was 10 minutes). All protocols had a 15-minute warm-up at 55 W. The dotted lines in the second row represent the sweat chloride concentration from conventional laboratory sweat tests

using Macroduct for sweat sample collection. The sweat sample collected at 200 W during the reverse trial was not sufficient for analysis (QNS).

In the single step trial, the sweat chloride concentration increased from 12 mM during the 100 W segment to 21 mM in the 200 W segment. During the reverse step trial, the sweat concentration at the end of 200 W was 34.8 mM and the concentration decreased down to 14.2 mM when the exercise load decreased from 200 to 100 W. The sweat sample during the 200 W segment was not sufficient for laboratory analysis. The concentration during the 100 W segment was 22 mM. During the multi-step trials (**Fig. S2C**), the concentration gradually increased and reached 35.8 mM at 200 W. The laboratory test results for the samples collected during the 100 to 150 W and 175 to 200 W segments were 12.5 and 27 mM, respectively. Other vitals also followed the changes in exercise load. The heart rate had a faster response than sweat profile. The maximum heart rates during the single, reverse, and multi-step trials were 182.4, 172.7, and 157.5 bpm, respectively. The core and skin temperature increased up to 37 ˚C at 200 W during the single and reverse step trials. The sweat rate also increased or decreased according to the exercise load. This subject's sweat rate increased from 0.12 to 0.30 μL min^{-1} cm^{-2} during the single step trial and decreased from 0.28 to 0.07 μL min^{-1} cm^{-2} during the reverse step trial.

Whole body sweat loss and forearm sweat loss.

The weight change before and after the trial was measured using a conventional scale (HBF-514C, Omron, 0.1 kg resolution) without clothing and after removing all sweat from their body. The whole body sweat loss was calculated based on a body surface area determined using the Du Bois formula. The forearm sweat loss was determined the Macroduct collection devices over the whole trial.

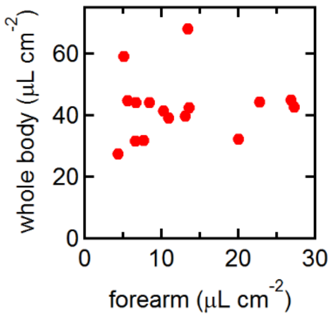

Figure S3. Whole body sweat loss and forearm sweat loss. Data shown for single step trials (N = 8) and reverse step trials (N = 9). While the whole body sweat loss is proportional to forearm sweat volume for some subjects, there is no correlation for many subjects.

A recent study of 11 healthy individuals while spinning at low intensity (45% $VO_{2,max}$) for 90 minutes showed a correlation between ventral forearm sweat rate and whole body sweat rate (r^2 = 0.91) [3]. Sweat collection was performed using absorbent patches. However, the authors noted that the fit did not have a slope of 1.0 (slope 0.4) and did not pass through the origin. The origin of the discrepancy between our results (Fig. S3) and this study is not known, although the sample size was small in both cases. Our trials involved at least three segments at different exercise intensity, which may also contribute to differences.

3. L. B. Baker. Physiology of sweat gland function: The roles of sweating and sweat composition in human health. *Temperature (Austin)* 6, 211-259 (2019).

Sweat rate following chemical sweat induction

We compared the sweat rates for 8 subjects during exercise and following chemically-induced sweating (see **Figure 6**). The sweat rates during exercise and following iontophoresis were obtained as described in the *Methods* section.

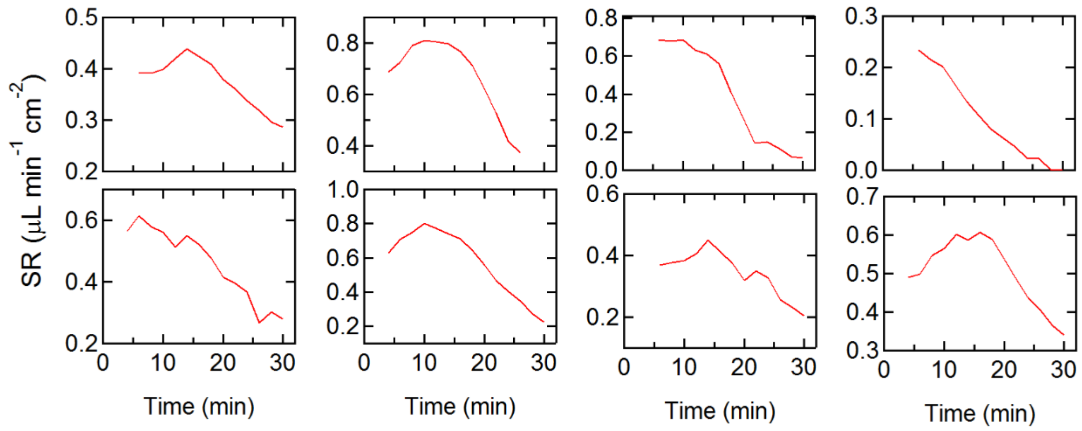

Figure S4. Sweat rate for 8 healthy individuals over time following pilocarpine iontophoresis.

Wearable sweat sensor

Sweat profiles were recorded using a potentiometric wearable chloride ion sensor developed in our lab (**Fig. S5A**). The salt bridge in the potentiometric sensor was designed to minimize equilibration between the sweat sample and the reference solution (1 M KCl) (**Fig. S5B-D**). The sensor output voltage was determined from calibration curves of the sensor voltage in standard solutions, with a slope of 52.8 ± 0.7 mV/decade (**Fig. S5E**). From long term tests with 100 µL of 10 mM NaCl, the drift rate due to equilibration was 0.3 mM h^{-1} over 12 hours (**Fig. S5F**).

Calibration of the sensors was performed prior to all measurements. All devices were calibrated in the following way: (1) the working electrode was rinsed in running deionized (DI) water for 40 s, (2) 100 µL of 10 mM NaCl (Fisher Scientific) solution was placed on the working electrode of the sensor using a micropipette, (3) the sensor voltage was measured and recorded for 3 minutes, (4) steps 1 - 3 were repeated with 50 and 100 mM NaCl solutions, (5) the sensor voltage for each solution was determined by averaging the recorded voltages over last 1 min, and (6) using a linear least squares fit (V-log C), the relationship between the measured voltage and the concentration of the test solution was established. All calibrations were performed at room temperature.

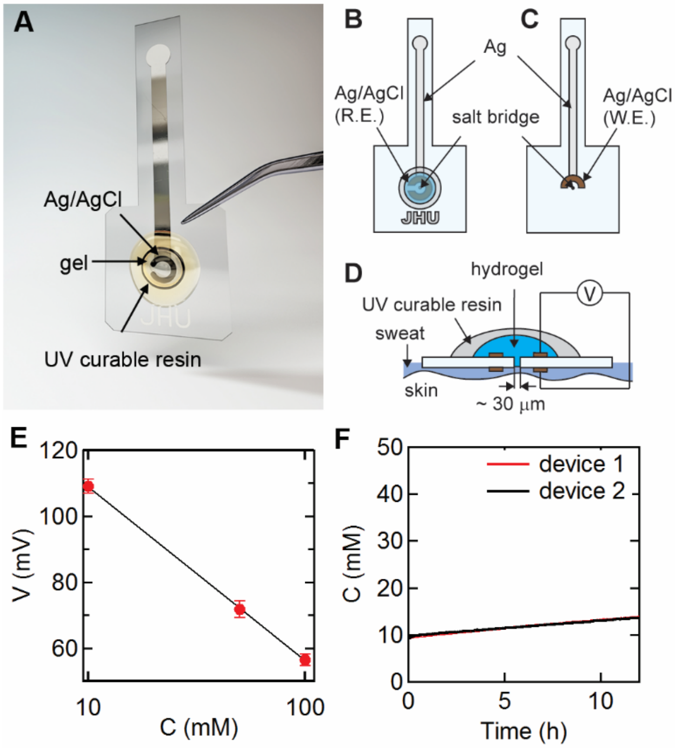

Figure S5. **Wearable sweat chloride sensor.** (A) Optical image of the sensor after fabrication. Schematic illustration of the sensor: (B) top side (reference electrode), (C) bottom side (working electrode), and (D) cross-section. (E) Sensor calibration curves (N = 6). Data represent mean ± SD. (F) Measured chloride ion concentration over 12 hours in 110 µL of 10 mM NaCl.
